# Supplementary material for: Direct Photochemical Synthesis of Substituted Benzo[b]fluorenes
Source: Org Lett. 2024 Nov 25;26(48):10364–8. doi: 10.1021/acs.orglett.4c03978 (PMC11629382; doi:10.1021/acs.orglett.4c03978)
Supplement: Supplementary file 2 — ol4c03978_si_002.zip [file ol4c03978_si_002.zip › FID for Publication/6d/20241008_RC-2-C-6-166B-P_01/plots/RC-2-C-6-166B-P_HSQCAD_20241008_01_plot01.pdf]

RC-2-C-6-166B-P

Location	**17**	Pulse sequence	**HSQCAD**	Temperature	**25**	Suboperator	**RuairiCrawford**
Date collected	**2024-10-08**	Solvent	**cdcl3**	Spectrometer	**localhost.localdomain-vnmrs500**	Operator	**MBaumann**

SAMPLE

date	**Oct 8 2024**
solvent	**cdcl3**
sample	**20241008_RC-2-C-6-166B-P_01**

ACQUISITION

sw	**5434.8**
at	**0.150**
np	**1630**
fb	**4000**
ss	**32**
d1	**1.000**
nt	**2**

2D ACQUISITION

sw1	**21361.8**
ni	**96**
phase	**arrayed**

PRESATURATION

satmode	**n**
wet	**n**

TRANSMITTER

tn	**H1**
sfrq	**499.721**
tof	**-259.6**
tpwr	**58**
pw	**6.300**

DECOUPLER

dn	**C13**
dof	**-2485.2**
dm	**nny**
decwave	**W40_P7374**
dmf	**32258**
dpwr	**43**
pwxlvl	**62**
pwx	**14.000**

HSQC

j1xh	**146.0**
nullflg	**y**
mult	**2**

FLAGS

hs	**nn**
sspul	**y**
PFGflg	**y**
hsglvl	**5736**

ADIABATIC

pwx180ad	**P7374_ad300**
pwx180adR	**P7374_ad300R**
pwx180	**465.4**
pwxlvl180	**57**
pwx180ref	**P7374_ref200**
pwx180r	**2000.2**
pwxlvl180r	**48**

SPECIAL

temp	**25.0**
gain	**40**
spin	**0**

F2 PROCESSING

gf	**0.069**
gfs	**not used**
fn	**2048**

F1 PROCESSING

gf1	**0.004**
gfs1	**not used**
proc1	**lp**
fn1	**2048**

DISPLAY

sp	**-478.4**
wp	**5434.8**
sp1	**-1257.5**
wp1	**21361.8**
rfl	**478.4**
rfp	**0**
rfl1	**1257.5**
rfp1	**0**

PLOT

wc	**142.6**
sc	**0**
wc2	**127.1**
sc2	**12.7**
vs	**48**
th	**3**
ai	cdc ph

ACQUISITION ARRAYS

array	**phase**
arraydim	**192**
i	phase
1	**1**
2	**2**

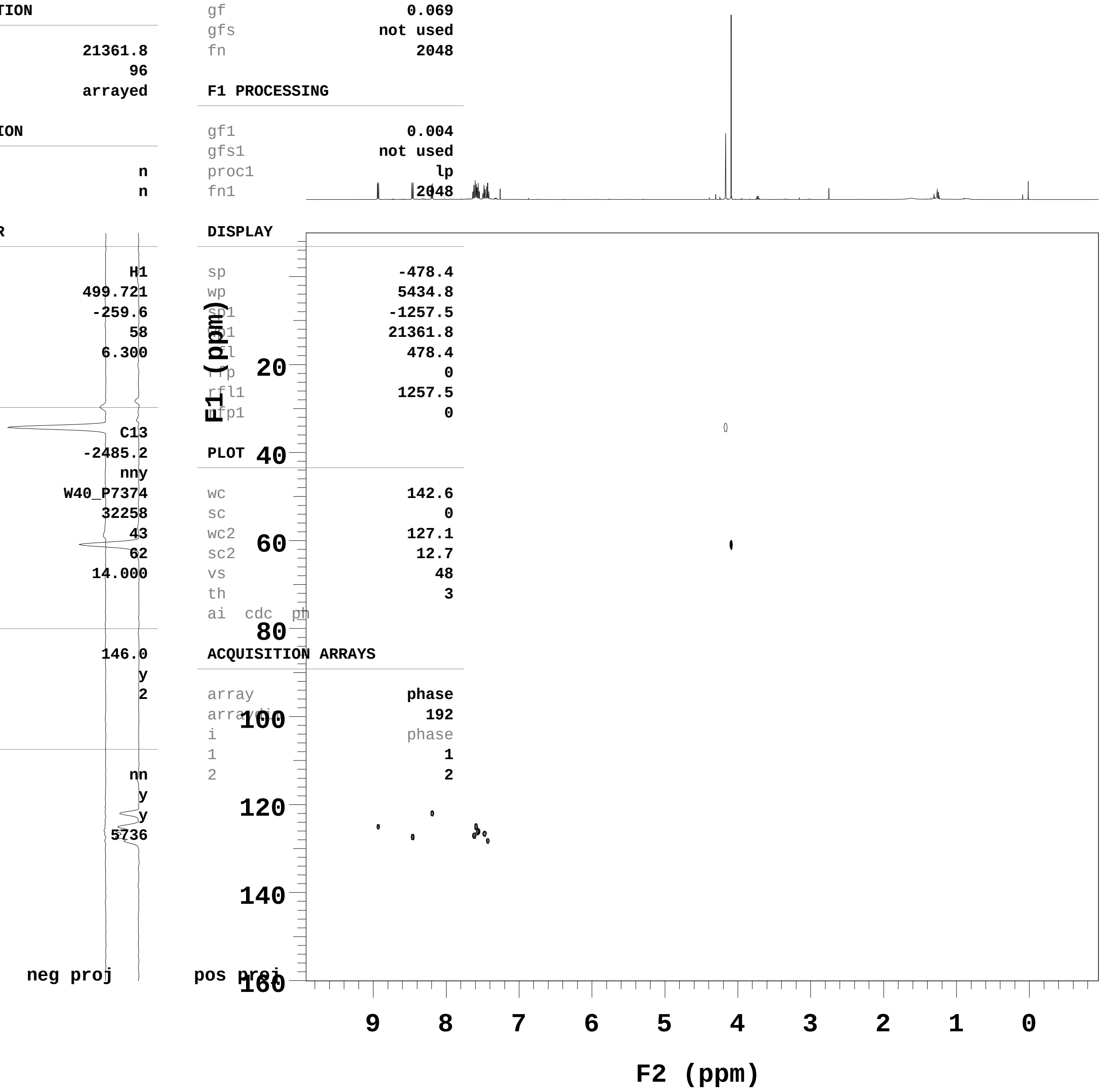

Plotname: RC-2-C-6-166B-P_HSQCAD_20241008_01_plot01

Data file /home/data/MBaumann/cscb500/RuairiCrawford/2024/20241008_RC-2-C-6-166B-P_01/RC-2-C-6-166B-P_HSQCAD_20241008_01.fid Plot date 2024-10-08
